# Impact of Whole Body Vibration and Zoledronic Acid on Femoral Structure after Ovariectomy: Morphological Evaluation

**DOI:** 10.3390/jcm11092441

**Published:** 2022-04-26

**Authors:** Nazar M. Kostyshyn, Izabela Świetlicka, Ewa Tomaszewska, Piotr Dobrowolski, Siemowit Muszyński

**Affiliations:** 1 Department of Normal Physiology, Danylo Halytsky Lviv National Medical University, Pekarska St. 69, 79010 Lviv, Ukraine; 2 Department of Biophysics, Faculty of Environmental Biology, University of Life Sciences in Lublin, Akademicka St. 13, 20-950 Lublin, Poland; izabela.swietlicka@up.lublin.pl (I.Ś.); siemowit.muszynski@up.lublin.pl (S.M.); 3 Department of Animal Physiology, Faculty of Veterinary Medicine, University of Life Sciences in Lublin, Akademicka St. 12, 20-950 Lublin, Poland; 4 Department of Functional Anatomy and Cytobiology, Maria Curie-Skłodowska University, Akademicka St. 19, 20-033 Lublin, Poland; piotr.dobrowolski@umcs.lublin.pl

**Keywords:** osteoporosis, ovariectomy, rat model, whole body vibration

## Abstract

Our study aimed to evaluate the effect of whole body vibration (WBV) treatment as an non-pharmacological method of treatment for early osteopenia in ovariectomized female rats. In total, 48 female Wistar rats were assigned to two groups: sham-operated control (SHAM, *n* = 12) and ovariectomized (*n* = 36). Four weeks after ovariectomy, the animals were divided into three experimental groups (*n* = 12 each): ovariectomized (OVX), ovariectomized subjected to whole body vibration with acceleration level of 0.3 g (OVX + WBV), or ovariectomized subjected to i.m. injection of Zoledronic acid at a dose of 0.025 mg/kg (OVX + ZOL). After the 8th and 16th week of treatment *n* = 6 rats from each group were euthanized and isolated femora were subjected to histological examination of trabecular bone and analysis of the expression of collagen 1 (Col1), osteoprotegerin (OPG), and receptor activator of nuclear factor kappa-Β ligand (RANKL) involved in bone turnover. The obtained results indicated that widespread vibration therapy can provide negative outcomes such as deterioration of trabecular bone histomorphometry.

## 1. Introduction

Bone is a complex, hierarchically organized system of structures that provides support and protection to the body [1,2,3]. The geometry and structure of bone are optimized in such a way as to minimize weight, dissipate the load on the joints and withstand external loads on the body [4,5,6]. In general, due to high rigidity, strength, elasticity and small weight, bone has a combination of mineral and protein components, as well as a hierarchical structure [6,7]. Normal bone metabolism involves a balance between bone resorption and bone formation, with osteoclasts resorbing bone and osteoblasts releasing a new organic matrix into the resorption cavity. It is believed that the basis for accelerating bone remodeling is a partial reduction in osteoblast life expectancy and prolongation of osteoclast life expectancy/activity, which, in turn, leads to osteoporosis [8]. The reason for this may be taking certain medications, calcium and vitamin D deficiency, low levels of sex hormones in menopausal women, or an interaction of these factors, which together contribute to the risk of osteoporotic fractures [2,9,10,11]. In postmenopausal women, bone loss increases rapidly and leads to osteoporosis. However, osteopenia and even osteoporosis with the risk of bone fracture can occur in young women after adnexectomy, polycystic ovaries, etc. [5,9,11,12].

Since osteoporosis is a systemic disease, it is accompanied not only by loss of bone mineral density, but also by structural disorders that lead to reduced strength, increased fragility and increased risk of fractures, especially of the femur and lumbar spine [2,5]. In addition to the mineral composition of bone nanocomposite and mineral density, it is important to study bone cells and the amorphous component—bone matrix [13,14]. The usual predictor of bone strength, bone mineral density (BMD), is used in vivo and is not linked to histological evaluation of bone matrix and its proteins. Histological evaluation allows one to assess proteins of the bone matrix, but is often limited to experimental conditions and therefore insufficiently studied. Therefore, preclinical studies in animal models of human diseases are still needed.

Drug therapy with anabolic hormones (parathyroid hormone (PTH)), antiresorptive therapy (bisphosphonates) and especially anti-receptor activator of nuclear factor kappa-Β ligand (RANKL) monoclonal antibodies (Denosumab) have beneficial effects in slowing bone loss [15,16,17,18,19,20,21,22]. However, these drugs have a number of contraindications, including nephrotoxicity, gastrointestinal intolerance, muscle atony and even osteonecrosis with pathological fractures [23,24,25,26]. Therefore, the search for non-pharmacological methods of prevention and/or treatment of osteopenia in people of different ages and various comorbidities remains an important issue [27,28,29]. It has been established that high-frequency vibration can be anabolic for the trabecular layer of the bone, both in animal models and in humans, and therefore can potentially increase the density and quality of bone tissue. Such methods can be promising, both for the treatment of osteopenia and for the prevention of bone loss in patients of different ages and with concomitant pathology [30,31,32,33,34].

The specific mechanisms by which mechanical stress stimulates bone formation are not fully understood. However, one of the factors that can affect the degree of bone formation caused by whole-body vibration is the ability to regulate the lifespan of osteocytes and inhibit osteoclast differentiation [35,36,37,38,39,40,41]. Vibration causes intracellular fluid fluctuations in osteocytes and osteoblasts, which leads to their activation through mechanically sensitive Piezo 1 ion channels, promoting bone growth [39,40,41,42,43,44,45,46]. In general, the magnitude of the bone formation reaction rises with increasing load, but we previously revealed that high vibration acceleration can have a devastating effect on the spinal microstructure, and vibration with an acceleration level of 0.05–0.13 g did not affect mineral density remodeling [47].

Rubin and McLeod [48] described in detail changes in bone tissue under the influence of mechanical stimuli, using an adaptation model of a turkey’s bone. Thus, the dependence of bone sensitivity on the frequency of mechanical stimuli was demonstrated [48]. Other authors showed that low-frequency mechanical vibration can effectively enhance trabecular bone formation: mature turkeys were fixed on a vibrating platform, oscillated at 30 Hz (0.3 g acceleration) for five minutes a day for 30 days. They investigated the marked surface of the trabecular bone of the proximal ends of the tibia and femur and showed a linear increase in mineral density depending on the speed of vibration acceleration [49,50].

The specific purpose of this study was to experimentally investigate the bone trabecular morphology and mechanisms of bone remodeling in ovariectomized rats under the influence of whole body vibration as a factor in antiresorptive therapy. The results are compared with the ones obtained for Zoledronic acid treatment, a bisphosphonate most often used for osteoporosis treatment in various pathologies, which can eliminate ovariectomy disorders in both trabecular and cortical bone. The results of this study can provide a better understanding of osteoporosis pathogenesis and form a new assessment of the vibration effectiveness as a new antiosteoporotic treatment in terms of improving the morphological state of the matrix and bone cells.

## 2. Materials and Methods

The study was performed in accordance with EU Directive 2010/63/EU and approved by the Local Ethical Committee No 10, 16 December 2019, Danylo Halytsky Lviv National Medical University in Lviv, Ukraine.

### 2.1. Animals and Experimental Design

**Figure 1:**
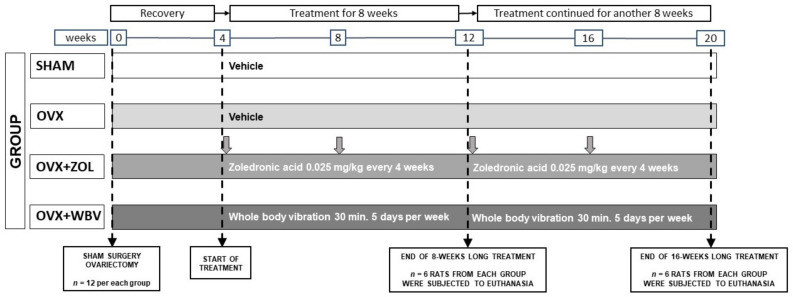
Scheme of the experimental design. Four weeks after the ovariectomy, rats from the OVX + WBV group were subjected to whole body vibration treatment (30 min., 5 days per week) while rats from the OVX + ZOL group were injected with Zoledronic acid (i.m. 0.025 mg/kg every 4 weeks, vertical gray arrows show the time of injections). Rats from the SHAM, OVX and OVX + WBV groups were injected with the vehicle (0.9% NaCl) at the same volume. After the 8th and the 16th week of treatment, six animals from each group were euthanized.

The experimental study was performed on 48 female Wistar rats aged 2 months and weighing 180–200 g. The animals were in a constant 12-h cycle of light and darkness, air temperature 21–23 °C and relative humidity 60 ± 10%. After one week of acclimatization, rats were randomly assigned to two groups: sham-operated control (SHAM, *n* = 12) and ovariectomized (*n* = 36). Four weeks after the surgery, ovariectomized rats were divided into three groups: untreated (OVX, *n* = 12), injected *i.m.* with Zoledronic acid at a dose of 0.025 mg/kg (OVX + ZOL, *n* = 12), or subjected to whole body vibration with an acceleration level of 0.3 g (OVX + WBV, *n* = 12) (Figure 1). Zoledronic acid (ZOLTA^®^, Amaxa Pharma, Liviv, Ukraine) was administered (quadriceps femoris) every 4 weeks. This route of administration was chosen due to technical availability and slow distribution of the drug. All other groups were injected i.m. with the same volume of physiological saline (ca. 6 µL) every 4 weeks. The rats in OVX + WBV groups were exposed to WBV for 30 min, 5 days per week. All experimental animals were weighed weekly to monitor their body weight. No changes were observed in the injection site on the skin and subcutaneous tissue. After the 8th and the 16th week, six animals from each group were euthanized under general intraperitoneal anesthesia [51].

### 2.2. Method of Ovariectomy in the Rats

The animals were fixed in the dorsal recumbent position. Before the surgery, 1 mL of 10% glucose solution was administered to prevent hypoglycemia. We used 0.3 g/kg of urethane solution for general intraperitoneal anesthesia. Lower median laparotomy and abdominal revision were performed. The horns of the uterus were removed with tweezers. Ligatures (4-0 Vicryl, Ethicon, Johnson & Johnson, New Brunswick, NJ, USA) were applied to the proper ovarian ligament between its bursa and the uterine horns through the mesovarium. The horns of the uterus and the mesovarium, which is rich in fat, were cut off to reduce infectious postoperative complications. The second ligature was applied dorsally to the ovarian bursa to ligate the ovarian artery and vein, then the ovary was excised and layered sutures were applied to the wound (PDS 3-0 Ethicon, Johnson & Johnson, New Brunswick, NJ, USA). One day after the laparotomy with ovariectomy, animals had a standard diet and were kept separately in individual plastic cages for seven days.

### 2.3. Whole-Body Vibration (WBV)

A treatment protocol was adapted from Xiao [52] with slight modifications [28,49]. Vertical vibration oscillations were modelled using a 250 W APC Rain-60 vibration pump (AquaPlanet Company, Ilfov, Romania) with the maximum pressure of 7 bar and a voltage regulator of the AFC-120 model (SophPower Electronics, Dongguan, China). A vibrating platform with the container, where the experimental group of rats was placed, was attached to the stem of the vibrating pump. The level of vibration acceleration was 3.0 m/s^2^ (0.3 g) with a frequency of 50 Hz and an amplitude of 1.2 mm. The rats were exposed to WBV constantly for 30 min (without interruptions), 5 days per week, which was similar to the protocol proposed by Flieger et al. [53]. During this weekly two-day break, rats were injected with the saline every 4 weeks to limit the effect of injection in the quadriceps muscle on vibration treatment.

### 2.4. Bone Preparation, PSR Staining, and Histomorphometry

Prepared fragments of bone tissue of the distal femur of experimental rats, cleaned from muscle tissue, were fixed in 10% formalin solution for 24 h in an airtight container for storage of biological material. After complete fixation, the preparations were washed with running water. Next, decalcification of bone tissue with a decalcifier solution (rapid descaler, Kaltek slr, Saonara, Italy) was performed for 48 h at a ratio of 1:10 at room temperature. The decalcified material was washed with distilled water and soaked in chloroform. The resulting bone material was filled with paraffin-wax mixture (3:1) and placed in a vacuum thermostat at 37 °C. Then, using a microtome (HM360, Microm, Walldorf, Germany), it was cut into 5-μm thick sections. Sections were stained with Picrosirus red (PSR). Stained sections were observed in normal light to assess basal histomorphometry of the trabecular bone in femur distal epiphysis and metaphysis: the relative bone volume (bone volume over the total volume), trabecular thickness, trabecular separation, and trabecular number. The percentage of immature collagen in PSR stained sections observed in polarized light (seen as green) was calculated using the pixel counting method as a proportion of total collagen content [54]. Microscopic images were collected using a light microscope (CX43, Olympus, Tokyo, Japan) and all analyses were performed using ImageJ software [55].

### 2.5. Immunohistochemical Staining

For immunohistochemistry (IHC), the following antibodies were diluted (1:100) in Diamond antibody diluent (Cell Marque Corp., Rocklin, CA, USA) and were used as primary antibodies (Abcam, Cambridge, UK): rabbit polyclonal against osteoprotegerin (OPG; ab73400), mouse monoclonal against receptor activator for nuclear factor kappa-B ligand (RANKL; ab239607), and rabbit polyclonal antibody against I collagen (Col1, ab34710). Negative control sections for each antibody were conducted by identical immunohistochemical staining excluding the primary antibody. The intensity of immunoreaction was measured using a CX43 microscope (Olympus, Tokyo, Japan) both by determining the percentage of cells with a positive response, and by the quantitative comparison of mean pixel intensity in the photomicrographs, which were first converted into negatives and then into 8-bit grey-scale digital images, with a scale from 0 (white pixel) to 255 (black pixel), where the higher the pixel value, the higher the intensity of the immunohistochemical reaction [56]. For the immunoreactive cell count procedure, four randomly selected areas of trabecular bone were measured for each microscopic slide and the percentage of positive immunoreactive cells was reported. The intensity of immunoreaction was measured in twelve randomly selected areas of the positive signal in trabecular bone and measured separately for cells (osteocytes) and the bone matrix. All the analyses were carried out blindly using ImageJ Software [55].

### 2.6. Statistical Analysis

The statistical analysis was performed using Statistica 13.1 (Palo Alto, CA, USA) and Origin2021b (OriginLab, Northampton, MA, USA). The sample size was calculated for a two-way ANOVA with an α of 0.05 and power at 0.8, with the number of groups equal to 8 (4 levels of treatment and 2 levels of time) [57]. With the above assumptions, the levels of 0.5 × SD between groups should be detected as significant. Literature data indicates that the sample size of *n* = 6 has a power of 80% to detect a change of 6% in BV/TV, 11% in Th.Sp and 13% in Tb.N [14], and 7% in Tb.Th [58] assuming a 5% significance level. Normality was assessed by the Shapiro-Wilk test, while the homogeneity of the variance was studied using Levene’s test. A two-way ANOVA, with the applied treatment and time as the main effects, was used to assess the observed changes and post-hoc Tukey’s test was applied to evaluate the groups’ differences in the analyzed parameters. Non-parametric data were analyzed using a Kruskal-Wallis H test. Additionally, a Dunnett’s test was performed to evaluate the differences between the experimental groups and the corresponding sham-operated controls (SHAM). Dunnett’s test was not performed when a lack of immunohistochemical responses was observed for the SHAM group. For all tests, a *p*-value < 0.05 was established as statistically significant. All the data are reported as mean ± standard error.

## 3. Results

### 3.1. Histomorphometry

The studies carried out in the area of the relative bone volume in the epiphysis showed the appearance of few significant changes observed for WBV or ZOL therapy following OVX (Table 1). The BV/TV ratio decreased after 16 weeks for both treatments, while after 8 weeks, a significant decrease was observed only for WBV following OVX. Mean and maximum trabecular thickness increased notably after ZOL treatment after 8 weeks following OVX, while after 16 weeks, a significant reduction of Tb.Th was observed when compared to the 8 weeks. In turn, WBV following OVX resulted in an increase in mean trabecular space after 8 weeks, while Tb.Sp_max_ and Tb.N remained unchanged irrespective of treatment (Table 1). Dunnett’s test of comparisons with the SAHM group showed that statistically significant differences appeared for the experimental groups for which the OVX was treated with WBV or ZOL. WBV following OVX resulted in a significant decrease in BV/TV and Tb.N, and a simultaneous increase in mean and maximum Tb.Sp and Tb.N after 8 weeks, while ZOL following OVX induced an increase of mean and maximum Tb.Th. On the contrary, after 16 weeks, significant reductions in BV/TV and mean and maximum Tb.Th were observed for both post-OVX therapies. The remaining parameters did not show differences compared to the corresponding SHAM groups.

Statistical analysis showed that the applied treatments (WBV or ZOL) caused changes in the histomorphometry of the metaphysis; however, the most notable ones for almost all investigated parameters were observed for WBV (Table 2), where histomorphometry examination revealed a decrease in BV/TV and Tb.N in relation to the SHAM group for both treatment periods. Consequently, an increase in the mean and maximum Tb.Sp was observed for WBV following OVX. However, WBV applied in the 8-week period did not change Tb.Sp_max_ in OVX subjects. Mean and maximum Tb.Th remained on a similar level irrespective of the implemented treatment (Table 2). Dunnett comparison with the corresponding SHAM group showed that OVX alone and combined with WBV and ZOL treatment influenced the BV/TV ratio, decreasing its value. Mean Tb.Sp was shown to be prone to OVX, after 16 weeks, irrespective of the applied treatment, while no changes were observed in the first 8-week-long period. Tb.Th_max_ values differed from the SHAM group only after 8 weeks and only for WBV following OVX. Furthermore, WBV caused significant alterations in mean and maximum Tb.Sp, inducing their increase, apart from the Tb.Sp_max_ for the 8-week period, which did not differ from the SHAM group. WBV following OVX also reduced the Tb.N number.

**Figure 2:**
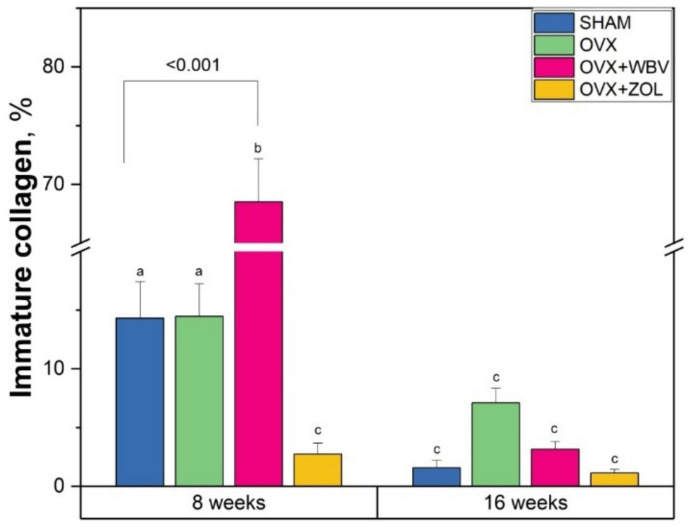
Immature collagen content in femora trabeculae of controls (SHAM surgery), ovariectomized rats (OVX), ovariectomized rats with whole body vibration (OVX + WBV), or Zoledronic acid (OVX + ZOL). Data are presented as mean ± SE. Statistically significant differences between groups are indicated by a, b and c letters as shown by two-way ANOVA with Tukey’s post hoc test (*p* < 0.05). Brackets show statistically significant results of Dunnett’s test post-hoc performed to evaluate the differences between the experimental groups and the corresponding sham operated controls (SHAM).

Immature collagen content increased significantly after 8 weeks due to WBV treatment when compared to both SHAM and OVX groups (Figure 2). At the same time, ZOL following OVX caused a significant decrease in immature collagen content. The immature collagen concentration after 16 weeks was significantly lower in SHAM, OVX and OVX-WBV groups when compared to the 8-week-long period and did not differ among groups.

### 3.2. Collagen 1 Immunoreaction in Femoral Bone

**Figure 3:**
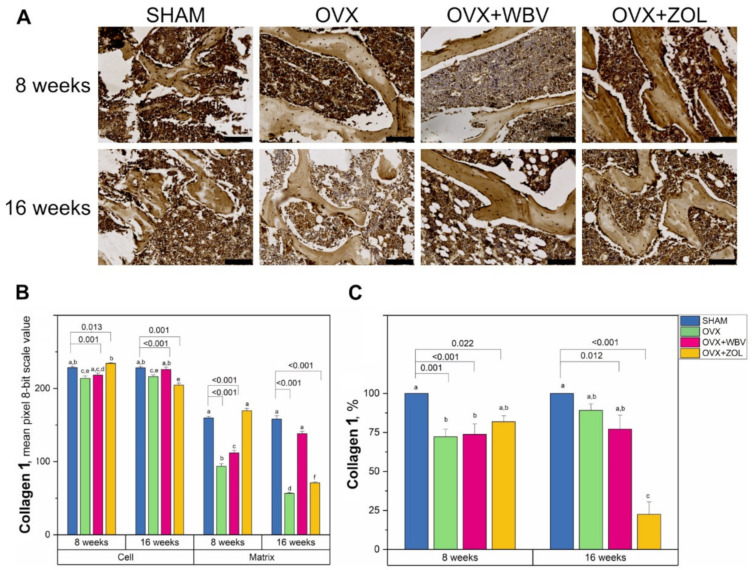
(**A**) Representative images of the immunohistochemical reactions for collagen 1 (Col1) in the trabecular bone of femora from controls (SHAM surgery), ovariectomized rats (OVX), ovariectomized rats with whole body vibration (OVX + WBV), or Zoledronic acid (OVX + ZOL). All the scale bars represent 100 μm. (**B**) Bar graphs show the intensity of expression of Col1 in cells (osteocytes) and matrix measured by the comparison of the pixel brightness value in the microscopic images converted to 8-bit grayscale—the higher the pixel value, the higher the intensity of the immunohistochemical reaction. (**C**) The percentage of cells with a positive response. Data are presented as mean ± SE with corresponding statistical analysis. Statistically significant differences between groups (at *p* < 0.05) are indicated by a, b, c, d, e, and f letters. Dunnett post-hoc significant *p*-values are placed over brackets.

Collagen 1 immunoreaction intensity in trabecular bone presented different values depending on the area and time period (Figure 3). The OVX after 8 weeks and ZOL following OVX after 16 weeks gave the lowest values of collagen immunoreaction intensities in bone cells. In the matrix, where generally lower intensities of reactions were observed, OVX with ZOL after 8 weeks and OVX alone in 8 and 16 weeks decreased the collagen 1 reaction intensity by about half compared to the SHAM groups. Dunnett’s comparison test with the sham-operated groups showed that OVX and OVX + WBV differed significantly from the corresponding SHAM group at 8 weeks, and OVX and OVX + ZOL for both the cell and matrix region after 16 weeks.

### 3.3. Osteoprotegerin (OPG) Immunoreaction in Femoral Bone

**Figure 4:**
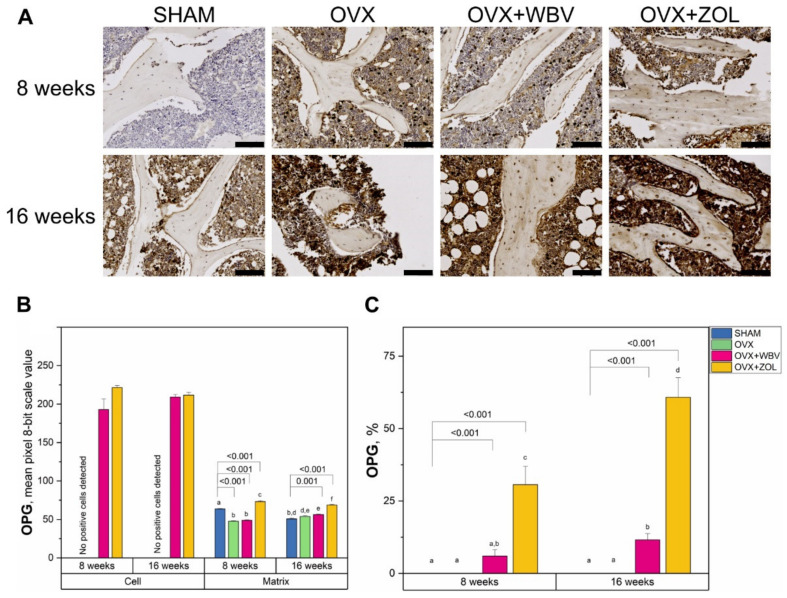
(**A**) Representative images of the immunohistochemical reactions for osteoprotegerin (OPG) in the trabecular bone of femora from controls (SHAM surgery), ovariectomized rats (OVX), ovariectomized rats with whole body vibration (OVX + WBV), or Zoledronic acid (OVX + ZOL). All the scale bars represent 100 μm. (**B**) Bar graphs show the intensity of expression of OPG in cells (osteocytes) and matrix measured by the comparison of the pixel brightness value in the microscopic images converted to 8-bit grayscale—the higher the pixel value, the higher the intensity of the immunohistochemical reaction. (**C**) The percentage of cells with a positive response. Data are presented as mean ± SE with corresponding statistical analysis. Statistically significant differences between groups (at *p* < 0.05) are indicated by a, b, c, d, e, and f letters. Dunnett post-hoc significant *p*-values are placed over brackets.

The intensity of OPG immunoreaction in bone cells was not observed for the SHAM and OVX groups for both time periods. Application of WBV or ZOL to the OVX group caused an increase, irrespective of time. It can be noted however, that a slightly stronger, but not statistically significant, positive reaction may have been registered for the OVX + ZOL group (Figure 4). A different pattern was observed for the matrix for both periods. The time variable was not considered statistically significant; however, the time ×group interaction proved to be important. After 8 weeks, a significant decrease in intensity of the positive reaction for OVX and OVX + WBV was observed, while for OVX + ZOL, a significant increase was registered. Measurement conducted after 16 weeks revealed an increase in intensities and the maximum level was observed for the OVX + ZOL group. The percentage of cells with a positive reaction was significantly higher only for OVX + WBV and OVX + ZOL during both experimental periods; however, a higher number of positive cells was achieved after 16 weeks. The Dunnett test showed that, in the matrix, only OVE did not differ from the 16-week SHAM group. When the percentage of positive responses was considered, it could be seen that OVX + ZOL and OVX + WBV differed from the corresponding SHAM groups in both examined time periods.

### 3.4. Receptor Activator of Nuclear Factor Kappa-Β Ligand (RANKL) Immunoreaction in Femoral Bone

**Figure 5:**
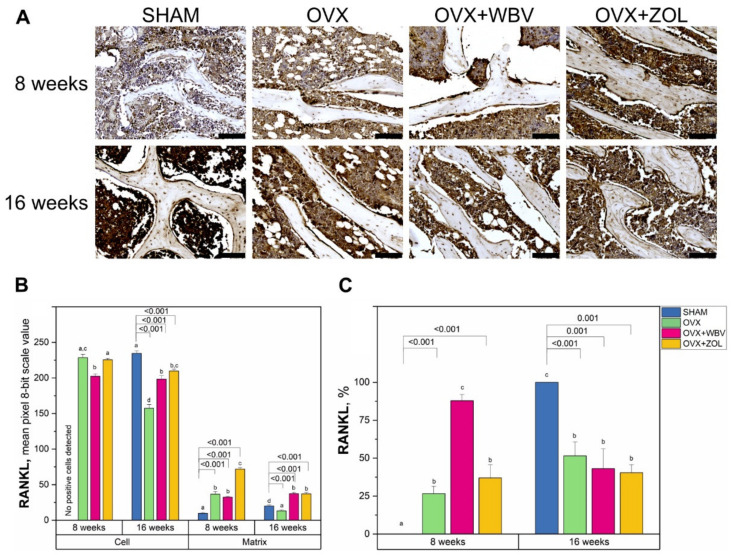
(**A**) Representative images of the immunohistochemical reactions for receptor activator of nuclear factor kappa-Β ligand (RANKL) in the trabecular bone of femora from controls (SHAM surgery), ovariectomized rats (OVX), ovariectomized rats with whole body vibration (OVX + WBV), or Zoledronic acid (OVX + ZOL). All the scale bars represent 100 μm. (**B**) Bar graphs show the intensity of expression of RANKL in cells (osteocytes) and matrix measured by the comparison of the pixel brightness value in the microscopic images converted to 8-bit grayscale—the higher the pixel value, the higher the intensity of the immunohistochemical reaction. (**C**) The percentage of cells with a positive response. Data are presented as mean ± SE with corresponding statistical analysis. Statistically significant differences between groups (at *p* < 0.05) are indicated by a, b, c, and d letters. Dunnett post-hoc significant *p*-values are placed over brackets.

The quantitative comparison of the intensities for the RANKL protein immunoreaction in cells showed that for the 8-week period, OVX, alone or with WVB/ZOL treatment, resulted in manifestation of RANKL positive cells, while for the SHAM group, no reaction was observed (Figure 5). In comparison, after 16 weeks, a significant decrease was observed. It was noticed that WBV following OVX gave similar results, irrespective of time. A rising trend for both periods might be observed when the matrix is considered. After 8 weeks, the most remarkable increase in the reaction intensity was registered for ZOL treatment following OVX. The changes observed after 16 weeks were not so significant, but the upward trend was still maintained. The only significant decrease was observed for OVX alone. The percentage of positive responses was significantly higher from the SHAM group after the 8-week period, while the highest values were registered for the OVX + WBV group. After 16 weeks, in turn, the percentage of positive responding cells was at a similar level for all ovariectomized groups and a significantly lower level than the corresponding SHAM group, and did not differ from the results in OVX or OVX + ZOL observed after 8 weeks. Dunnett’s’ test showed that all the experimental groups differed significantly from the corresponding SHAM groups.

## 4. Discussion

The main goal of this experiment was to investigate whether the whole body vibration has the same beneficial effect as standard drug therapy with bisphosphonates in the case of bone loss in ovariectomized rats. The bisphosphonate Zoledronic acid was chosen as one of the drugs most often used for osteoporosis treatment in various pathologies. Zoledronic acid, as monotherapy, is known to improve bone mineral mass and increase hydroxyapatite, which can eliminate ovariectomy disorders in both trabecular and cortical bone. Bisphosphonates have been shown to provide an additional advantage in maintaining mean BMD and have a positive effect on cortical bone geometry, while stabilizing collagen fibers and hydroxyapatite crystals with fewer bone pores, which may play an important role in preventing mineral loss.

There is enough information in the literature on the action of various drugs that can improve the quality and quantity of the mineral crystalline component, thereby increasing the mineral density of different parts of the skeleton. However, little is known about the morphometric parameters of trabecular bone and the cell response, as the state of the protein matrix is important for maintaining parameters such as elasticity and strength. Xiao [52] proposed a combination treatment for osteoporosis caused by ovariectomy. The author of the study hypothesized that the bisphosphonate ibandronic acid did not block the anabolic effect of PTH in the shins of ovariectomized rats when used in combination. With the right ratio of anabolic and antiresorptive drugs, the effect may be more pronounced. These results provide valuable information on the potential of the combination drug in osteoporosis treatment. However, it should be noted that the most favorable ratio of the two drugs was not optimized in this study [52]. Therefore, it is important to conduct further research to optimize the relationship between anabolic and antiresorptive therapy in order to improve the effects of treatment from simultaneous use.

It should be noted that the study model was chosen by us because of the frequent development of osteoporosis during menopause in women. The results of morphometry showed a significant difference between sham-operated (SHAM surgery) and ovariectomized groups of animals, which indicates the successful simulation of osteoporosis in rats. Such models of rats with ovariectomy have been widely used to study the effectiveness of osteoporosis treatment and compared to other animal models, it has a number of advantages: short study time and low cost of the experiment. However, there are differences in the structure of the cortical bone between rats and humans, in particular, the lack of remodeling in the haversian canals in rats [59,60]. Thus, the results obtained in such studies may not be consistent with clinical observations and therefore were not used in our study. However, better understanding of the pathogenesis of the remodeling process will be achieved in animal models with a well-developed osteone structure, namely rabbits, dogs, sheep, turkeys, etc. Furthermore, differences in age and different skeletal areas studied complicate comparison of the research results. It should be noted that Liu et al. [61] reported that a significant deterioration in the density and structure of the trabecular layer was found in tubular bones, lumbar vertebrae and iliac bone after OVX, while jaw bones remained relatively resistant to mineral loss by the 36th week [61]. Other authors, in particular Kim et al. [62], aimed to find a critical time for impaired trabecular bone regeneration after OVX in rats. Thus, a perforated defect was created on both sides of the upper jaw in castrated females and the OVX group showed significantly lower new bone formation and mineral density than other groups, as confirmed by micro-CT and histomorphometric analysis. Moreover, in this experimental group, bone metabolism increased significantly, which was confirmed by bone markers in the blood. The authors concluded that the critical time for impaired trabecular bone regeneration was 4 months after OVX in rats. Kakihata et al. [63] evaluated the effect of non-physiological vibration on the femur in sterilized rats. Vibration was applied for 10 min/day, with a frequency of 60 Hz, 3 d/week. OVE groups showed a decrease in spongy and cortical bone tissue. In addition, vibration therapy lasting 4 to 8 weeks in groups of ovariectomized animals led to an increase in bone mass, as evidenced by an increase in the percentage of spongy tissue, as well as an increase in the thickness and percentage of cortical tissue [63]. Other researchers, such as Hashimoto et al. [64], hypothesized that whole-body vibration (WBV) promotes fracture healing. After ovariectomy in mice, a through hole was created in the bone, after which they were subjected to WBV (30 Hz and amplitude 0.3 g). WBV treatment was administered for 30 min/day and 5 days/week. WBV tended to increase the volume fraction of defected bone and promote its regeneration [64]. In 2018, Runge et al. [65] treated rats with vibration with acceleration values from 0.15 g to 1.2 g, and recorded an increase in the density of cancellous bone [65]. Contrary to the above results, in our previous studies we recorded signs of acute damage to the bone tissue of the vertebrae and thighs starting with 0.56 g [47].

To understand the morphological changes of bone tissue, bone morphometry was performed. Morphometric changes of the trabecular bone are more noticeable in the epiphysis than the metaphysis during the 8th week of the experiment. Under the influence of vibration, thickening of bone trabeculae in the bone epiphysis was observed and similar changes were noticed in the group treated with Zoledronic acid. However, no significant effects on the morphometric characteristics of the femur were found in the metaphysis due to 8-week-long low-frequency vibration. Our study showed a mechanically dependent mechanism of action on the trabecular bone of the femur. The positive effect of WBV treatment was obvious, as evidenced by data in the relative bone volume (BV/TV) during the 8th week of study. The positive effect of WBV treatment was also observed in changes in the trabecular bone microarchitecture, and was particularly evident compared to ovariectomized females. All of the results discussed above may suggest that WBV should be used in the early post-ovariectomy or menopausal period, which is consistent with the study of Fliegier et al. [53], where a similar treatment protocol was applied and the most positive changes in femoral BMD were observed in the first 5 weeks of the WBV treatment. On the contrary, in the recent study of Kakihata et al. [63], the opposite effects were reported, as after eight-week-long WBV treatment, an increase in BV/TV in the femoral proximal epiphysis was observed. However, they applied a different treatment protocol, where the WVB treatment (60 HZ) was applied for 10 min, 3 d per week.

During the 16 week-long period of the experiment, our results showed that changes in bone morphometry were observed during the simulated artificial menopause, which may indicate accelerated remodeling; however, no statistically significant changes were recorded. It is obvious that during menopause the bones become weaker and more prone to fractures. In general, the trabecular bone is characterized by a higher rate of bone remodeling than compact bone, and therefore, any changes are initially observed in the histomorphometry of trabeculae. However, it should be noted that ovariectomy did not affect the thickness of the trabeculae in the epiphysis or metaphysis during the 8th week of the experiment. However, during the 16th week, we observed a rapid thinning of the trabeculae in this group. Such changes were also present in the OVX-ZOL group. This can be explained by the inhibitory effect of Zoledronic acid on bone metabolism. Therefore, its use should probably be limited to longer breaks between drug administrations. Prolonged vibration (16 weeks) caused a sharp degradation of the organic component in the trabeculae, probably due to impaired remodeling of the trabecular layer. However, in our previous studies, we observed a comparable level of hydroxyapatite crystalline fraction of mineral component of the trabecular bone [47].

We conducted immunohistochemical study of the bone, as its mechanical properties depend not only on the quantitative and qualitative composition of the mineral component [47]. Quantitative indicators of bone include mineral density and size of nanocomposites, which together form bone mass. However, in addition to BMD and nanocrystalline structure, the quality of the protein component of bone is also a decisive factor in determining bone strength. The combination of two different components in bone, in particular elastic fibrous collagen and the brittle mineral phase, results in a very strong and light material. The generalizations and functions of such material have aroused wide interest in the scientific community. With increasing attention to bone nanocomposites, various studies have been conducted to identify the features of their organic and inorganic components. It then became obvious that the density of mineral bone does not determine its strength in general [6,7,14,51]. Information on the basic structural unit of bone, i.e., mineral–collagen composite, will provide a better understanding of the volumetric behavior and fragility of tissue.

The described bone changes depend on the intensity of bone metabolism, which in turn depends on the activity of osteoblasts and osteoclasts. The main factors regulating this process are osteoprotegerin (OPG), a factor that inhibits osteoclastogenesis secreted by osteoblasts; RANKL, a glycoprotein produced by mature osteoblasts and their precursors that activate the process of formation of mature osteoclasts; and the RANK glycoprotein present on osteoclast precursors [66]. As a result of RANK–RANKL activation, the maturing osteoclast undergoes structural and metabolic changes that initiate a resorption effect. OPG can bind to RANKL as it is a ligand that prevents RANKL from binding to RANK and therefore stops osteoclasts from maturing. Differentiation, maturation and metabolic activity of osteoclasts, and hence the intensity of bone resorption, depend on the relative balance between RANKL and OPG levels. When RANKL exceeds OPG, the rate of bone resorption is pathologically increased, and when OPG exceeds RANKL, the intensity of the process of bone tissue loss is pathologically reduced [66,67].

Immunohistochemical analysis of the rat’s femur in the current study showed that treatment with Zoledronic acid or WBV has a significant effect on the level of collagen and non-collagen proteins in bone tissue in OVX rats. Although, the OPG positive cells were detected only in OVX + WBV and OVX + ZOL, the immunoreaction was observed in all groups in the matrix, and a significant increase was noted in the OVX + ZOL group. However, the most important for bone remodeling is RANKL production by osteocytes. Interestingly, the anabolic response of the bone to mechanical stimulus does not require living osteocytes [68]. In our study, the highest number of RANKL positive cells after 8-week-long treatment was observed in WBV group.

On the other hand, our study also showed that bisphosphonate stimulated bone cells to produce OPG and inhibited RANKL synthesis, as indicated by the percentage of OPG and RANKL positive cells compared to whole body vibration treatment. This decrease in the number of RANKL positive cells with reduced intensity can confirm the benefit of remodeling slowdown when using both whole body vibration or bisphosphonate. This effect, according to the literature, slows the loss of bone mineral component [69].

As mentioned above, the RANK/RANKL/OPG system is very important for bone remodeling and maintenance of bone mineral mass [70]. Our study showed that changes in OPG and RANKL synthesis depended on the duration of WBV treatment and the use of Zoledronic acid. For WBV, this effect was observed only for 8-week-long treatment as evidenced by RANKL and immature collagen 1 levels, while for Zoledronic acid this effect was still observed after 16-week-long treatment. The observed increase in OPG reaction in the OVX + ZOL group may be a positive effect on bone metabolism, given that OPG is central to the regulation of bone-related pathology. The balance between RANKL and OPG, as important factors determining the number and activity of osteoclasts, serves as a useful biomarker of bone turnover [71]. The intensity of OPG and RANKL reactions in rats in the current study showed that this balance is not always possible to track in the applied menopause model, although the overall OPG positive expression reaction may indicate beneficial effects of mechanical vibrations, especially at the 8th week of study, which is consistent with the general role of mechanical stimuli in RANKL/RANK/OPG related bone remodeling [72].

Collagen 1 plays an important role in the remodeling of bone trabeculae, as it is necessary for the deposition of hydroxyapatite and bone calcification. The rapid decrease in collagen 1 content as shown by IHC at the 16th week in the OVX + ZOL group is likely to indicate stabilization/retardation of bone remodeling and increased deposition of hydroxyapatite, which results in the increase in bone mineral density.

In our research, we studied the structure of the femoral bone tissue, as this area is of greatest interest for orthopedic research because it is in this area that osteoporotic fractures most often occur. A common cause of accelerated remodeling, and therefore loss of BMD, is menopause. Estrogen deficiency leads to accelerated bone metabolism and bone loss in humans [3,4,9,12]. Physiological mechanical stimulation in the form of physical activity and regular exercise can slow bone loss and even cause it to increase [33,34,39]. Evidence from previous studies suggests that although exercise is very effective in developing bone mass in adolescence, it has less effect in adults and the elderly [30]. It should be noted that a number of studies indicate that skeletal areas farther from the vibration source will have a smaller increase in the trabecular layer of bone than those located closer, probably due to a decrease in vibration because of the distance from the vibration source [40].

The detailed mechanism of whole-body vibration therapy, which has recently emerged as a non-pharmaceutical means for the treatment of osteoporosis, is not yet fully studied and understood. However, it is known that osteocytes are in the bone matrix, experience changes in mechanical stress, and produce signals that alter bone formation by osteoblasts [43,44]. Li et al. [73] reported that the Piezo1 ion channel, which is required for changes in gene expression caused by fluid shift stress in cultured osteocytes and stimulation by a small molecule Piezo1 agonist, Yoda 1, is sufficient to reproduce the effects of fluid flow on osteocytes [73]. The authors found that conditioned deletion of Piezo1 in osteoblasts and osteocytes markedly reduced bone mass and strength in mice. Conversely, administration of the Piezo1 agonist Yoda 1 to adult mice increased bone mass, mimicking the effects of mechanical stress. These results demonstrate that Piezo1 is a mechanically sensitive ion channel through which osteoblast lineage cells sense and respond to changes in mechanical stress and identify a new target for anabolic bone therapy.

For the purposes of our study, we chose vibration parameters with a safe frequency and low vibration acceleration, in order to avoid side effects from other tissues. We were guided by the ISO Standards for General Vibration [35,36] (ISO 2631-1, 1997 and ISO 2631-5, 2004), which stated a safe level of 20–90 Hz and a vibration acceleration level of less than 0.56 g. According to these data, such fluctuations do not lead to acute or chronic tissue damage and have anabolic and/or anticatabolic effects on bone tissue. However, such vibration parameters should be used with caution, due to the ability to resonate with tissues and organs of the human body [37,38].

Whole body vibration (WBV) as a method of treatment can be used passively, especially for people with limited mobility. In addition, it uses the natural response of the bone to the load, and therefore does not carry potential side effects that drug treatments may have [32,36]. However, the question of whether non-pharmaceutical methods will help treat bone during physiological menopause and reduce the risk of fractures remains open. Despite the positive effects of vibration, it is not able to affect the underlying cause—estrogen deficiency—although it has a positive effect on bone, especially in the early postmenopausal period. Due to conflicting data, the pathophysiological relation between obesity, sedentary lifestyle, and loss of BMD is complex and needs further study.

In addition, we studied the morphological parameters of bones using WBV on ovariectomized rats and compared them with the results of the group with the additional use of Zoledronic acid. Taking into account the reports of many authors on the high rates of osteoformation and increase in BMD in long bones and vertebrae of animals with ovariectomy after WBV [61,62,63,74,75], we assume that this effect was observed with long-term use and possibly in the remote period.

However, no significant effects from 4 months of low-frequency vibration (50 Hz, 0.3 g) on the morphometric characteristics of the femur were found. There are several possible explanations that this expectation did not come true. It should be noted that although the effect of WBV on the femur microstructure in the study data was significant, in general, the effect of WBV on the bone microstructure and BMD, as shown, varies depending on the location, type and parameters of vibration (magnitude, frequency, duration). In fact, rats treated with WBV had significantly lower lumbar BMD than the group that did not receive such treatment. In addition, the maximum vertebral load was significantly reduced in WBV-treated rats [47].

The obtained results allowed us to hypothesize that non-physiological vibration with an acceleration level of 0.3 g is effective in bone loss prevention during early postovariectomy, which was studied in an animal model. The vibration acts on the points of the body contact with the vibrating platform and is transmitted from the feet of the subject through the rest of the body. It is likely that this vibration is somewhat attenuated in the lower body, so the skeletal areas that are further away are likely to experience less vibration than the areas that are close to the ground. An important and beneficial effect of WBV treatment is that it significantly reduces excess body weight in postmenopausal osteoporosis. This result indicates that widespread vibration therapy cannot provide a therapeutic effect under all conditions; on the contrary, negative results may occur with a particular WBV treatment regimen. This conclusion suggests a review of WBV use for the prevention and treatment of osteoporosis. Based on our results, we believe that WBV should not be used as a safe treatment for all patients with osteoporosis. As the optimal parameters are still being studied, WBV should not be recommended for long-term use. The overall results of this study provide a better understanding of osteoporosis pathogenesis and provide a new assessment of the effectiveness of various osteoporotic drugs (drug combinations as well as whole-body vibrations). Our study expands the conclusions of others about the application of whole-body vibration [53,63,76,77].

The limitation of this study is that it is difficult to control the amount of vibration that reaches the pelvis and the possible resonance phenomenon. We observed that during WBV the behavior of rats did not differ from the SHAM group. It is also likely that the amount of vibration experienced at different skeletal sites is not constant. More proximal areas of the skeleton are likely to receive less vibration due to soft tissue dumping prior to vibration. In addition, the direction of the load on different skeletal areas varies depending on posture. In the future, we are planning to study the combination of drugs and WBV with different parameters to establish the minimum effective dose of the drugs and assess their impact in different parts of the skeleton. Further, it is necessary to measure BMD and histomorphometry for in-depth analysis of other bones to study the risk of fractures in different parts of the skeleton; to analyze the effect of other (smaller) doses of Zoledronic acid on bone metabolism, as well as the combined effect of vibration with bisphosphonates.

## 5. Conclusions

Based on the results of the current rat model study, it can be assumed that the use of WBV 0.3 g for 8 weeks has a positive effect on bone, although this effect is significantly inferior to traditional treatment with bisphosphonates. Furthermore, widespread vibration therapy can provide negative outcomes such as deterioration of trabecular bone histomorphometry. However, there is a need to further explore the possibility of additional use of whole-body vibration for prophylactic or therapeutic purposes in humans for osteoporosis treatment.

## Figures and Tables

**Table 1:** The trabecular bone histomorphometry in femur epiphysis in the study groups.

Factor		BV/TV, %		Tb.Th, mm		Tb.Th_max_, mm		Tb.Sp, mm		Tb.Sp_max_, mm		Tb.N, 1/mm	
Time	Treatment	Mean ± SE	Dunnett	Mean ± SE	Dunnett	Mean ± SE	Dunnett	Mean ± SE	Dunnett	Mean ± SE	Dunnett	Mean ± SE	Dunnett
8 weeks	SHAM	30.5 ± 2.4 ^abc^	-	28.5 ± 3.4 ^a^	-	51.4 ± 4.5 ^a^	-	56.0 ± 3.9 ^a^	-	89.6 ± 3.9	-	11.0 ± 0.8	-
	OVX	29.4 ± 1.2 ^bc^	0.995	27.6 ± 1.1 ^a^	0.999	56.5 ± 6.8 ^a^	0.997	65.3 ± 6.7 ^ab^	0.908	98.0 ± 8.5	0.945	10.7 ± 0.6	0.999
	OVX + WBV	24.4 ± 1.2 ^c^	0.032	31.7 ± 2.8 ^ab^	0.931	56.0 ± 12.6 ^a^	0.999	85.0 ± 7.6 ^b^	0.004	118.8 ± 9.34	0.043	7.9 ± 0.5	0.021
	OVX + ZOL	35.3 ± 1.6 ^bc^	0.147	43.8 ± 3.2 ^bc^	0.002	77.5 ± 9.1 ^b^	0.023	62.4 ± 3.7 ^ab^	0.996	92.7 ± 7.2	0.999	8.2 ± 0.6	0.049
16 weeks	SHAM	36.9 ± 1.5 ^a^	-	47.3 ± 2.9 ^c^	-	86.7 ± 11.4 ^b^	-	72.1 ± 3.3 ^ab^	-	108.1 ± 6.0	-	7.9 ± 0.3	-
	OVX	35.0 ± 1.3 ^ab^	0.907	43.8 ± 4.0 ^bc^	0.904	58.7 ± 0.9 ^a^	0.049	60.4 ± 3.8 ^a^	0.880	91.3 ± 2.9	0.441	8.4 ± 1.1	0.992
	OVX + WBV	28.8 ± 1.0 ^bc^	0.003	35.6 ± 1.1 ^abc^	0.024	60.0 ± 4.9 ^a^	0.051	77.8 ± 6.1 ^ab^	0.317	104.9 ± 9.3	0.999	8.1 ± 0.4	0.999
	OVX + ZOL	26.6 ± 1.0 ^c^	<0.001	28.5 ± 1.9 ^a^	<0.001	58.2 ± 6.5 ^a^	0.048	58.7 ± 2.8 ^a^	0.611	85.7 ± 9.0	0.177	9.7 ± 1.1	0.335
ANOVA *p*-value													
Main factors													
Time		<0.001		0.495		0.021		<0.001		0.056		0.128	
Treatment		0.075		0.004		0.332		0.846		0.664		0.068	
Interaction													
Time × Treatment		<0.001		<0.001		0.054		0.178		0.153		0.088	

Results are presented as mean ± SE with corresponding statistical analysis. SHAM—sham operated control group, OVX—ovariectomized rats, OVX + WBV—ovariectomized rats + whole body vibration, OVX + ZOL—ovariectomized rats + Zoledronic acid treatment. BV/TV—bone volume over the total volume, Tb.Th—trabecular thickness, Tb.Th_max_—maximum trabecular thickness, Tb.Sp—trabecular space, Tb.Sp_max_—maximum trabecular space, Tb.N—trabecular number. Statistically significant differences between groups (at *p* < 0.05) are indicated by ^a^, ^b^ and ^c^ superscript letters as shown by two-way ANOVA with Tukey’s post hoc test. Dunnett—*p*-values (significant at *p* < 0.05) of Dunnett’s post-hoc test performed to evaluate the differences between the experimental groups and the corresponding sham operated controls (SHAM).

**Table 2:** The trabecular bone histomorphometry in femur metaphysis in the study groups.

Factor		BV/TV, %		Tb.Th, mm		Tb.Th_max_, mm		Tb.Sp, mm		Tb.Sp_max_, mm		Tb.N, 1/mm	
Time	Treatment	Mean ± SE	Dunnett	Mean ± SE	Dunnett	Mean ± SE	Dunnett	Mean ± SE	Dunnett	Mean ± SE	Dunnett	Mean ± SE	Dunnett
8 weeks	SHAM	36.0 ± 3.3 ^ab^	-	25.6 ± 2.5 ^a^	-	52.7 ± 7.4 ^ab^	-	46.8 ± 3.9 ^a^	-	80.6 ± 6.8 ^a^	-	14.1 ± 0.4 ^a^	-
	OVX	26.1 ± 1.8 ^ac^	0.041	25.1 ± 3.4 ^a^	0.999	43.8 ± 6.2 ^a^	0.865	63.3 ± 7.0 ^a^	0.152	95.0 ± 10.0 ^ab^	0.482	10.9 ± 1.0 ^abc^	0.041
	OVX + WBV	24.4 ± 0.8 ^c^	0.013	26.6 ± 1.3 ^a^	0.999	49.0 ± 3.1 ^ab^	0.999	75.2 ± 5.3 ^b^	0.003	98.8 ± 3.6 ^ab^	0.251	9.3 ± 0.5 ^bc^	0.001
	OVX + ZOL	28.4 ± 0.9 ^ac^	0.049	27.4 ± 0.9 ^a^	0.992	51.3 ± 4.8 ^ab^	0.999	56.5 ± 2.2 ^a^	0.644	87.2 ± 3.0 ^a^	0.969	10.4 ± 0.5 ^abc^	0.015
16 weeks	SHAM	43.4 ± 2.7 ^b^	-	44.2 ± 3.7 ^b^	-	75.3 ± 10.0 ^b^	-	53.5 ± 2.5 ^a^	-	82.3 ± 4.8 ^a^	-	10.0 ± 0.5 ^bc^	-
	OVX	33.1 ± 3.9 ^abc^	0.031	33.2 ± 1.4 ^a^	0.012	60.1 ± 5.5 ^ab^	0.390	64.7 ± 6.3 ^a^	0.506	100.8 ± 5.8 ^ab^	0.235	10.2 ± 1.5 ^bc^	0.999
	OVX + WBV	23.9 ± 1.4 ^c^	<0.001	30.3 ± 2.3 ^a^	0.001	52.7 ± 4.2 ^ab^	0.082	89.8 ± 6.8 ^b^	<0.001	118.0 ± 8.3 ^b^	0.003	8.0 ± 0.5 ^c^	0.401
	OVX + ZOL	33.7 ± 3.2 ^abc^	0.048	26.4 ± 1.7 ^a^	<0.001	60.8 ± 7.9 ^ab^	0.440	50.0 ± 5.6 ^a^	0.997	73.8 ± 6.9 ^a^	0.898	12.8 ± 1.0 ^ab^	0.106
ANOVA *p*-value													
Main factors													
Time		<0.001		0.010		0.171		<0.001		<0.001		0.001	
Treatment		0.009		<0.001		0.006		0.282		0.476		0.113	
Interaction													
Time × Treatment		0.369		0.001		0.484		0.244		0.115		0.004	

Results are presented as mean ± SE with corresponding statistical analysis. SHAM—sham operated control group, OVX—ovariectomized rats, OVX + WBV—ovariectomized rats + whole body vibration, OVX + ZOL—ovariectomized rats + Zoledronic acid treatment. BV/TV—bone volume over the total volume, Tb.Th—trabecular thickness, Tb.Th_max_—maximum trabecular thickness, Tb.Sp—trabecular space, Tb.Sp_max_—maximum trabecular space, Tb.N—trabecular number. Statistically significant differences between groups (at *p* < 0.05) are indicated by ^a^, ^b^ and ^c^ superscript letters as shown by two-way ANOVA with Tukey’s post hoc test. Dunnett—*p*-values (significant at *p* < 0.05) of Dunnett’s post-hoc test performed to evaluate the differences between the experimental groups and the corresponding sham operated controls (SHAM).

## Data Availability

The data presented in this study are available on request from the corresponding authors.
